# Early Cognitive and Behavioral Deficits in Mouse Models for Tauopathy and Alzheimer’s Disease

**DOI:** 10.3389/fnagi.2019.00335

**Published:** 2019-12-06

**Authors:** Celine Samaey, An Schreurs, Stijn Stroobants, Detlef Balschun

**Affiliations:** ^1^Brain and Cognition, KU Leuven, Leuven, Belgium; ^2^Center for Clinical Psychiatry, KU Leuven, Leuven, Belgium; ^3^Leuven Brain Institute, KU Leuven, Leuven, Belgium

**Keywords:** Alzheimer’s disease, tauopathy, transgenic mouse model, early symptoms, preclinical, cognition, social behavior

## Abstract

Neurocognitive disorders, among which Alzheimer’s disease (AD), have become one of the major causes of death in developed countries. No effective disease-modifying therapy is available, possibly because current treatments are administered too late to still be able to intervene in the disease progress. AD is characterized by a gradual onset with subclinical neurobiological and behavioral changes that precede diagnosis with years to even decades. The earlier the diagnosis, the earlier potential treatments can be tested and started. Mouse models are valuable to study the possible causes underlying early phases of neuropathology and their reflection in behavior and other biomarkers, to help improve preclinical detection and diagnosis of AD. Here, we assessed cognitive functioning and social behavior in transgenic mice expressing tau pathology only (Tau-P301L) or a combination of amyloid and tau pathology [amyloid precursor protein (APP)-V717I × Tau-P301L]. The mice were subjected to a variety of behavioral tasks at an age of 3–6 months, i.e., at an early phase of their AD-like pathology. We hypothesized that compared to age-matched wild-type controls, transgenic mice would show specific impairments in both cognitive and non-cognitive tasks. In line with our expectations, transgenic mice showed decreased cognitive flexibility in the Morris water maze, decreased exploratory behavior, decreased performance in a nesting task, and increased anxiety-like behavior. In accordance with the amyloid-cascade hypothesis, some of the behavioral measures showed more severe deficits in APP-V717I × Tau-P301L compared to Tau-P301L mice, indicating an exacerbation of disease processes due to the co-occurrence of amyloid and tau pathology. Our study supports the use of behavioral markers as early indicators of ongoing AD pathology during the preclinical phase.

## Introduction

Alzheimer’s disease (AD) is the most common form of dementia, accounting for over 70% of all cases (Jaworski et al., 2010), and is estimated to affect over 40 million people worldwide (Selkoe and Hardy, 2016). AD is a complex, heterogeneous disease with a variable age of onset, rate of progression, and development of pathology. The disease is characterized by two major neurobiological hallmarks: extracellular amyloid plaques and intracellular neurofibrillary tangles (NFTs) containing hyperphosphorylated tau (Spires-Jones and Hyman, 2014).

Amyloid plaques are mainly composed of Aβ_40_ and Aβ_42_ amino acid polypeptides that are derived from the amyloid precursor protein (APP) by proteolytic cleavage. APP is cleaved at three cleavage sites, the α-, β-, and γ-secretase site, leading to the release of Aβ peptides and other APP fragments of different lengths. Especially Aβ_42_ is neurotoxic, as it has a high propensity to form oligomeric aggregates and eventually deposit as plaques. Transgenic mice expressing Aβ_42_ develop amyloid plaques, whereas Aβ_40_-mice do not (Götz and Ittner, 2008). Moreover, Aβ_40_ seems to have a protective function, as it prevents Aβ_42_ from aggregating and forming plaques (Götz and Ittner, 2008). In general, Aβ levels rise because of both increased production and impaired elimination of Aβ_42_ (Jaworski et al., 2010). NFTs are formed by aggregated tau, a microtubule binding protein that is mainly found in axons, where it stabilizes microtubuli and likely plays a role in cellular transport processes (Spires-Jones and Hyman, 2014). Tau is a phosphoprotein and its activity is tightly regulated by phosphorylation (Iqbal et al., 2010). In AD and other tauopathies, tau is hyperphosphorylated and dissociates from the microtubuli, consequently forming NFTs (Götz and Ittner, 2008). Tau is hypothesized to have prion-like seeding characteristics and occurs in different conformational strains, which have been linked to distinct tauopathies (Sanders et al., 2014; Kaufman et al., 2016), but the clinical relevance of this hypothesis remains to be further clarified (Mudher et al., 2017).

The amyloid cascade hypothesis, still the dominant model for AD pathogenesis, explains the progression of pathology and the genetic risk factors that underlie both familial AD (FAD) and the much more common sporadic AD (SAD) (Karran et al., 2011). It states that it is the amyloid pathology that initiates the pathological cascade of AD, with tau pathology as a downstream effect (Mufson et al., 2016; Selkoe and Hardy, 2016). Functional deficits like synaptic dysfunction, neuronal cell death, and dementia are already apparent, and partially reversible in animal models, at intermediate stages of the cascade (Sydow et al., 2011; Ahmed et al., 2015; Krüger and Mandelkow, 2016). Several studies have demonstrated that Aβ plays a critical role in the development of AD, for example, increases in the Aβ_42_/Aβ_40_ ratio predispose individuals to develop AD (Karran et al., 2011). Furthermore, there is evidence that tau pathology arises downstream of amyloid pathology. For instance, amyloid pathology may actually induce tau pathology through the activation of GSK-3 isozymes (Terwel et al., 2008). In addition, injection of Aβ_42_ fibrils induced an increase in the number of NFTs in Tau-P301L mice, whereas the reverse was not observed (Götz et al., 2001), providing further evidence for the hypothesis that amyloid pathology precedes NFTs. However, this direct causality is incompatible with clinical observations and some recent studies support the view that the Aβ–tau interaction is not exclusively unidirectional (De Strooper and Karran, 2016).

Alzheimer’s disease progresses gradually from mild to severe dementia and eventually death, with a mean survival of 10 years after diagnosis (American Psychiatric Association, 2013). The disease is defined by early memory deficits, especially in episodic memory, and gradual deterioration of other cognitive functions, including problems with working memory, executive functioning and language, object use and/or recognition, confusion in time and place, as well as verbal fluency problems (Götz and Ittner, 2008; Webster et al., 2014). In addition to these cognitive deficits, AD is also characterized by several behavioral and psychological signs and symptoms of dementia (BPSD), which place a high burden on caregivers and the patient’s family. Examples of these BPSD are changes in personality, deterioration of social skills, social withdrawal, emotional dulling, depression, aggression, behavioral disinhibition, and psychosis (Jaworski et al., 2010; Lyketsos et al., 2011). Furthermore, patients with AD also display disturbances in diurnal rhythm and altered sleep–wake patterns (Volicer et al., 2001).

Alzheimer’s disease is often preceded by a period of mild cognitive impairment (MCI), a clinical condition in which cognitive decline is greater than expected given a person’s age and educational level, but does not interfere with activities of daily living (ADLs) (Gauthier et al., 2006). During MCI, several cognitive deficits arise: impairments in executive functioning, attention, and visuospatial memory, followed by impairments in verbal recall and finally impairments in general cognition (Webster et al., 2014). In addition, MCI is also commonly associated with increased neuropsychiatric symptoms, especially depression and anxiety (Palmer et al., 2007; Lyketsos et al., 2011). Most people with MCI remain stable or even improve over time, but one-third to half of all people with MCI progress to AD within 5 years (Gauthier et al., 2006; Fischer et al., 2007; Palmer et al., 2007). Neuropsychiatric symptoms in MCI, especially depression, apathy, and anxiety, are a predictor of progression to AD (Palmer et al., 2007; Teng et al., 2007).

Even before MCI, small changes in cognition and behavior can occur, which are considered preclinical changes. For example, impairments in episodic and semantic memory already arise near the end of the preclinical phase (Webster et al., 2014), with longitudinal studies describing significant impairments in episodic memory 10–12 years before symptom onset in patients with FAD (Bateman et al., 2012) and SAD (Amieva et al., 2008). Furthermore, in a study of cognitively normal older adults, people with positive AD biomarkers, more specifically cerebrospinal fluid (CSF) and neuroimaging biomarkers, performed worse on all cognitive measures than people without AD biomarkers (Hassenstab et al., 2016). A meta-analysis highlighted significant preclinical deficits in episodic memory as well as in global cognitive ability, perceptual speed, executive functioning, verbal ability, visuospatial skills, and attention (Bäckman et al., 2005). Moreover, initial personality changes and difficulties in attentional and inhibitory control were shown to be prominent before clinical diagnosis of AD (Balsis et al., 2005; Storandt, 2008). Late-life psychiatric symptoms, especially depression and anxiety, often precede MCI and AD (Steenland et al., 2012; Donovan et al., 2018). Finally, even subtle changes in instrumental ADLs (IADLs) can be observed 10 years before the official diagnosis (Fuentes, 2012).

The knowledge of neurobiological factors and genes underlying AD was largely non-existent until 25 years ago. Since then, animal models, especially transgenic mouse models, have been developed (Khachaturian, 2005). These models have been instrumental in the increasing knowledge on AD mechanisms and the development of potential therapeutics (LaFerla and Green, 2012). However, no animal model can fully replicate human AD pathology nor cognitive deficits. As a consequence, different animal models have been introduced for different research questions (Webster et al., 2014).

Although several potential disease-modifying drugs have been proposed for AD, there is still no cure for the disease. Many authors have attributed this lack of effect to the severity of the disease at treatment initiation (Emery, 2011; Counts et al., 2017). Consequently, early detection and diagnosis of AD is crucial for the development of treatments that might delay or even prevent AD. Therefore, this study aimed to identify early behavioral and cognitive deficits in transgenic mouse models. We opted for two littermate mouse models: one mutant for human Tau-P301L (Terwel et al., 2005) and a bigenic model expressing human APP-V717I × Tau-P301L (Terwel et al., 2008). The combined APP × Tau model suffers amyloid pathology from 10–12 months and tau tangles from 13 months in brain regions relevant for human AD, whereas the Tau-P301L mice show tau tangles from 6 to 9 months and die earlier (Terwel et al., 2005, 2008). In young mice on the FVB/N background, no overt pathological lesions were observed, but deficits in some cognitive tests (novel object recognition, passive inhibitory avoidance) and synaptic function were reported in males of 4–6 months (Terwel et al., 2008; Chong et al., 2011), while 1–2 month-old male Tau-P301L mice interestingly showed *enhanced* cognition and synaptic function (Boekhoorn et al., 2006; Kremer et al., 2011). Here, we were interested to investigate in more detail what happens at these young ages in these models (and now on C57BL/6J background). Although the Tau-P301L mutation has only been found in frontotemporal dementia patients, in mice it causes a tauopathy that contains 4R tau inclusions, the same as in human AD (Mudher et al., 2017). The mutation is therefore used in many other mouse models for AD [e.g., in the popular 3 × Tg-AD developed by Oddo et al. (2003)] and in studies investigating tau propagation in an AD context (e.g., De Calignon et al., 2012). Furthermore, direct comparison of the Tau and APP × Tau models allows to probe the potential additive effects of Aβ on tau pathology. Behavioral read-outs from these models can reveal early, prodromal changes that could be translated into markers for early detection of preclinical frontotemporal dementia or AD in humans. In combination with other biomarkers, these behavioral markers could enable the identification of people in the earliest disease stages, long before diagnosis, when drug interventions may be a lot more effective (Counts et al., 2017). Furthermore, identification of early functional parameters would allow a more sensitive evaluation of therapeutic efficacy in future preclinical studies using these and other mouse models.

## Materials and Methods

### Animals

Tau-P301L (Tau or TPLH) and APP-V717I × Tau-P301L (APP × Tau or biAT) mice were originally created on FVB/N background by Terwel et al. (2008). These models were recently backcrossed to C57BL6/J background by reMYND nv (Heverlee, Belgium), who kindly provided founder transgenic mice. All wild-type and transgenic mice used in this study were bred and housed in the animalium of the Laboratory of Biological Psychology, KU Leuven. Animals were group-housed under standard conditions (constant temperature and humidity; normal 12 h light/dark cycle; lights on at 8 a.m.), with *ad libitum* access to food and water.

Our sample consisted of 38 male mice, all on C57BL/6J background and with an average age (mean ± *SD*) of 11 ± 2 weeks at the start of testing, of which 13 wild-type, 13 Tau, and 12 APP × Tau mice. Throughout all behavioral tests, the experimenter was blind to the genotype of the animals. All animal experiments were approved by the KU Leuven Ethical Committee and in accordance with the European Directive 2010/63/EU.

### General Neuromotor Assessment

To gain insight in general exploration, diurnal pattern of locomotor activity, motor function, general arousal, and spontaneous activity were monitored every 30 min during 23 h (D’Hooge et al., 2005). Mice were individually placed in transparent home cages filled with 400 ml bedding and with modified cage tops that prevented them from climbing. Activity was recorded using three infrared beams and the Mouse4Win program.

Furthermore, the accelerating rotarod, as described before by D’Hooge et al. (2005), was used to evaluate motor coordination. During the training phase, mice were placed on the rotarod for 2 min, followed by 3 min of resting. The testing phase consisted of four trials, each maximally lasting 5 min on the accelerating rod, during which the rotation speed progressively increased from 4 to 40 r/min. Latency to fall was assessed during each trial. In between test trials, mice were given at least a 5-min break, since the inter-trial interval was 10 min.

### Natural Murine Behavior

Making nests is natural murine behavior, since they are important for thermoregulation, reproduction, and shelter. Moreover, research has shown that hippocampal lesions are associated with decreased nesting behavior, which can be interpreted as deterioration in the ability to perform ADLs, a key symptom of AD (Deacon, 2012). Therefore, nesting behavior was assessed in two tasks using distinct types of nesting materials.

Animals were placed in individual cages filled with 0.5 cm of normal bedding and a single paper towel as nesting material. After 23 h, the quality of the paper nests was assessed on a five-point scale (Deacon, 2012). A largely untouched nest with over 90% of material still intact was given a score of 1. Two points were given for a partially torn-up nest, with 50–90% intact. When less than 50% of the material was intact without identifiable nest site, a score of 3 was given. A four-point nest was defined by a clearly identifiable but flat nest site, whereas a five-point nest needed to have a nearly perfect crater with walls higher than the animal’s body height on 50% of the circumference. Exemplar pictures of the different scores are presented in Supplementary Figure S1.

The nesting was repeated with cylindrical 2 cm nesting material (Cocoon), which is more comparable to the Nestlets used by Deacon (2012), instead of paper, exactly 50 days after the first nesting task. The same scoring system as before was applied (Deacon, 2012). Example images of nests evaluated with the different scores are presented in Supplementary Figure S2.

### Exploration and Anxiety Assessment

Four tests were included to assess the level of exploration and anxiety-like behavior: the elevated plus maze, open field task, sociability and preference for social novelty (SPSN) test, and marble burying.

#### Elevated Plus Maze

Briefly, the elevated plus maze has two arms enclosed by walls and two open arms without walls (D’Hooge et al., 2005). Entries in both the closed and the open arms, as well as percentage of time spent in the open arms, were recorded by infrared beams. Each mouse was placed in the left closed arm of the maze and after 1 min of habituation, exploratory activity was recorded for 10 min.

#### Open Field

During the open field task (Naert et al., 2011), mice were placed in a brightly illuminated transparent plexiglas arena (50 × 50 × 30 cm) inside an enclosed cupboard. After 1 min of habituation, open field exploration was monitored during 10 min with ANY-maze tracking software (Stoelting Co., Wood Dale, IL, United States). More specifically, the total distance traveled, the average distance from the center, as well as the number of entries to, time spent in, and percentage distance traveled in the center, corner zones, and periphery were measured.

#### SPSN

The three-step SPSN protocol, consisting of a 5-min acclimation phase and two 10-min testing phases, was used to assess sociability and social memory (Naert et al., 2011). A transparent plexiglas box (94 × 28 × 30 cm) with three chambers separated by divider walls was placed in a closed cupboard with dim lighting. During the acclimation phase, the animals could move freely in the central chamber (29 × 28 × 30 cm). Empty cylindrical wire cages were present in the left and right chambers (36 × 28 × 30 cm each). In the sociability trial, the second phase of this test, a stranger mouse was placed in the wire cage in either the left or right chamber while the other wire cage remained empty. The chamber in which this stranger mouse was presented was alternated between test mice. The test animal was placed in the central chamber and allowed to explore all chambers freely. Finally, in the preference for social novelty trial, a second stranger mouse was placed in the empty cage, while the first stranger animal remained in the same location as in the previous trial. The test mouse was again placed in the central chamber and could explore all chambers freely. Explorative behavior, more specifically distance traveled, was recorded using ANY-maze video tracking. Additionally, time spent sniffing a stranger’s cage was scored manually during the trials. The stranger mice were group-housed C57BL/6J males that had already served as stranger mice in other SPSN experiments. Every stranger mouse was used only once per day.

#### Marble Burying Task

Finally, during the marble burying task, mice were placed individually in a large cage containing a 5 cm thick layer of standard bedding and 22 glass marbles distributed equally along the cage walls at approximately 2 cm distance from each other. The animals were left undisturbed in a quiet room for 30 min, after which the number of marbles that were buried with bedding for at least two-thirds were counted.

### Spatial Learning and Memory

The Morris water maze was used to assess hippocampus-dependent spatial learning and memory as described before (D’Hooge et al., 2005). The 150 cm circular pool was filled with opacified water at 25 ± 1°C and contained a hidden escape platform (15 cm). The EthoVision system (Noldus, Netherlands) was used to track the mice while in the pool. During the acquisition phase, each mouse was placed inside the pool four times a day from a different starting position, with an inter-trial interval between 15 and 30 min. Mice were gently guided to the platform if they failed to find it within 120 s. Two acquisition blocks were performed, each from Monday to Friday (5 days), followed by a pause during the weekend, and a probe trial on each Monday thereafter (referred to as days 6 and 11). During probe trials, the escape platform was removed from the pool. The mice were placed opposite of the former platform position and tested for 100 s. Finally, during the third week, the escape platform was moved to the quadrant opposite of the original position. Each mouse was subjected to four reversal trials per day, always starting from different positions, and this during 5 days. After the weekend, on day 16, retention of spatial information was tested again using a probe trial.

### Depression-Like Behavior

The tail suspension test is one of the most widely used tests to assess depression-like behavior and effectiveness of antidepressants in mice (Cryan et al., 2005). The mice were suspended by their tails with tape in a suspension box for 6 min. Their escape-oriented behaviors were video-recorded (ANY-maze, Stoelting Co., Wood Dale, IL, United States) and scored (Can et al., 2012). Immobility, the dependent variable, was measured in several ways: average distance from the center, the latency to immobility, total immobile time, and number of immobile episodes.

### Aversive Learning and Pain Perception

#### Fear Conditioning

Context- and cue-dependent fear conditioning was studied using a 3-day protocol as described before by Naert et al. (2011). During test day 1, animals were placed inside the test chamber (context A: dark room, grid floor, unscented) and allowed to adjust for 5 min. Their mobility was recorded using a force transducer with a sampling rate of 50 Hz (MED Associates Inc., St. Albans, VT, United States); 24 h later (test day 2), animals were again placed in the same test chamber (context A) for fear conditioning. After 2 min of acclimatization, two auditory cues (4 kHz, 80 dB) were administered for 30 s with a 1 min inter-stimulus interval. The auditory cue co-terminated with a 2 s long 0.2 mA foot shock administered through the grid floor of the test chamber. During these 3 min, shock-induced freezing was monitored. Another 24 h later (test day 3), during the contextual fear phase, the animals were returned to the test chamber (context A) for 5 min of exploration. At least 90 min later, the animals were again placed in the test chamber, but now in an altered context (context B: brightly illuminated, white plastic sheet covering the grid, scented with peppermint oil). Animals could explore this new context for 3 min, after which the conditioned stimulus (i.e., auditory cue) was presented during another 3 min. Animal movements were recorded by the force transducer and converted into freezing percentages. We calculated the discrimination index, defined as the difference between the percentage of freezing in the training context and the new context, divided by the sum of the two percentages, as a quantification of fear memory generalization (Xu and Südhof, 2013). In addition to the standard protocol, a fear retention test was included. Hereto, the animals were again tested in both context A (contextual fear phase) and context B (cued fear phase), exactly 21 days after the previous testing phase (referred to as test day 4).

#### Tail Withdrawal Test

The tail withdrawal test evaluates the ability of an animal to detect nociceptive stimuli, more specifically heat, and was used to rule out differences in pain perception, which can directly cause differences in conditioned fear responses. In order to reduce handling restraint and variability in handling by the experimenter, as well as to reduce stress, the mice were slightly restricted by entering a plastic cylinder voluntarily. Mice were placed in a custom made plastic restrainer, which allowed to lower the distal half of the tail in warm water. This procedure was performed twice at four increasing temperatures (47, 49, 51, and 53°C, respectively) as previously described (Leo et al., 2008). The latency to respond to this heat stimulus by flexing the tail strenuously was measured manually using a stopwatch. Animals were removed from the water immediately after responding or after a 25 s cut-off time to avoid tissue damage. Between same-temperature trials, an interval of at least 15 min was adopted. The between-temperatures interval was at least 30 min.

### Statistical Analyses

Statistical analysis of the open field, SPSN, and tail suspension tests was done using the analysis tools provided by ANY-maze (Stoelting Co., Wood Dale, IL, United States). More specifically, an ANOVA was performed with genotype as independent variable for the open field test; genotype and test phase for SPSN; and genotype and test segment as independent variables for the tail suspension test. *Post hoc* Tukey tests were executed in case of significant effects. RStudio version 1.1.463 (RStudio Team, United States) was used for statistical analysis of all other behavioral measures, using the R-packages *stats* and *lme4*. For the nesting task, elevated plus maze, marble burying task, and tail withdrawal test, a one-way ANOVA was applied to the raw data, or to transformed data when the assumptions of normality, homoscedasticity, or independence were violated. In case of robust violations of these assumptions, a non-parametric Kruskal–Wallis test, or one-way ANOVA on ranks, was performed. Datasets with multiple data points for each animal, i.e., spontaneous activity, rotarod, Morris water maze, and fear conditioning, were analyzed using linear mixed-effects models. All graphs were prepared using *ggplot2* in RStudio.

## Results

### General Neuromotor Assessment

#### Spontaneous Activity

**FIGURE 1:**
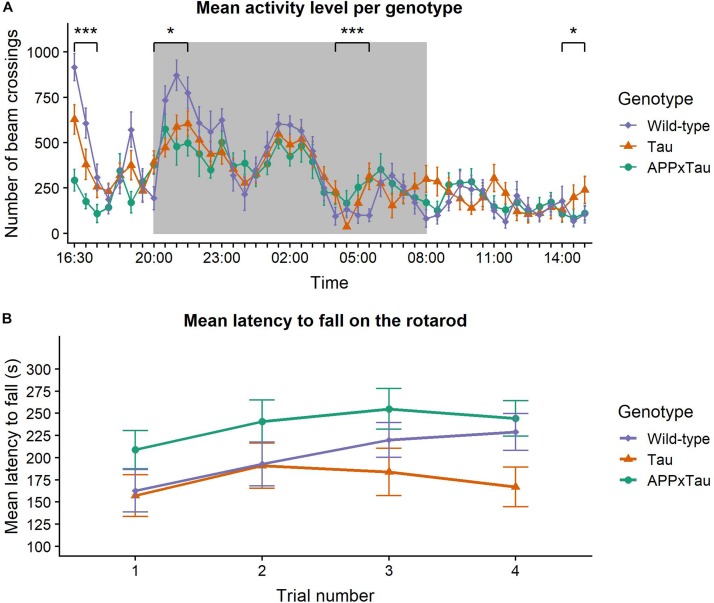
Neuromotor assessment. **(A)** Mean pattern of spontaneous activity during a 23-hour recording period. Time of the day had a significant main effect on the activity level (*p* < 0.001) and there was a significant interaction effect between time and genotype (*p* < 0.001). Wild-type animals were more active during the habituation phase and the first nocturnal peak, but these differences disappeared in later nocturnal peaks and the day phase. During the final time segment, Tau animals were more active than wild-types. The dark background indicates the night period in the lab. **(B)** Mean latency to fall on the rotarod. Linear mixed-effects model showed a significant effect of trial number on the latency to fall (*p* = 0.0141). The effect of genotype was marginally non-significant (*p* = 0.0576). Data are presented as mean + the standard error of mean. Note that the *y*-axis starts at 100 for clarity. ^∗^*p* < 0.05; ^∗∗∗^*p* < 0.001.

Results of the spontaneous activity measure are depicted in Figure 1A. A linear mixed-effects model indicated a main effect of time on the activity level of the animals (*F*_45_,_1581__.__1_ = 19.0941, *p* < 0.001), but no effect of genotype (*F*_2_,_35_ = 1.0957, *p* = 0.3455). There was, however, a significant interaction effect between time and genotype (*F*_90_,_1581__.__1_ = 2.358, *p* < 0.001). Overall, wild-type mice were more active during the habituation phase and at the first nocturnal peak, yet this difference declined during the second and third nocturnal peak and the light phase. Dunnett’s test was used for comparing spontaneous activity over a period of 23 h between transgenic and wild-type mice. There was a significant difference between APP × Tau and wild-type mice (*p* = 0.0066), but not between Tau and wild-type animals (*p* = 0.3923).

To gain insight in group differences over the course of 23 h, the time segments were analyzed individually. There was a significant effect of genotype during the habituation phase (16:30–18:00; χ^2^ = 25.751, *p* < 0.001), with wild-type animals showing significantly more activity than Tau and APP × Tau mice (*p* = 0.0084 and *p* < 0.001, respectively). There was also a significant effect during the first nocturnal peak (20:00–22:00) and immediately after the second nocturnal peak (04:00–06:00; χ^2^ = 6.1902, *p* = 0.04527 and χ^2^ = 15.415, *p* < 0.001, respectively). More specifically, during the first nocturnal peak, wild-type were more active than APP × Tau (*p* = 0.0475). *Post hoc* tests showed a significant difference between wild-type and both Tau and APP × Tau (*p* = 0.0255 and *p* < 0.001, respectively) after the second nocturnal peak. Finally, there was a significant genotype effect during the last time segment (14:00–15:30), when Tau showed increased activity compared to wild-type animals (χ^2^ = 6.2478, *p* = 0.044).

#### Rotarod

Figure 1B illustrates the mean latency to fall on the accelerating rotarod. A linear mixed-effects model revealed a significant effect of trial number on the latency to fall (*F*_3_,_104__.__97_ = 3.6993, *p* = 0.0141): motor coordination improved over time with significant differences between trial 1 and trials 2, 3, and 4 (*p* = 0.0272, *p* = 0.0101, and *p* = 0.0069, respectively). However, the effect of genotype was marginally non-significant (*F*_2_,_35_ = 3.1000, *p* = 0.0576). There was no interaction effect between trial number and genotype (*F*_3_,_104__.__97_ = 0.6836, *p* = 0.6632).

### Natural Murine Behavior

**FIGURE 2:**
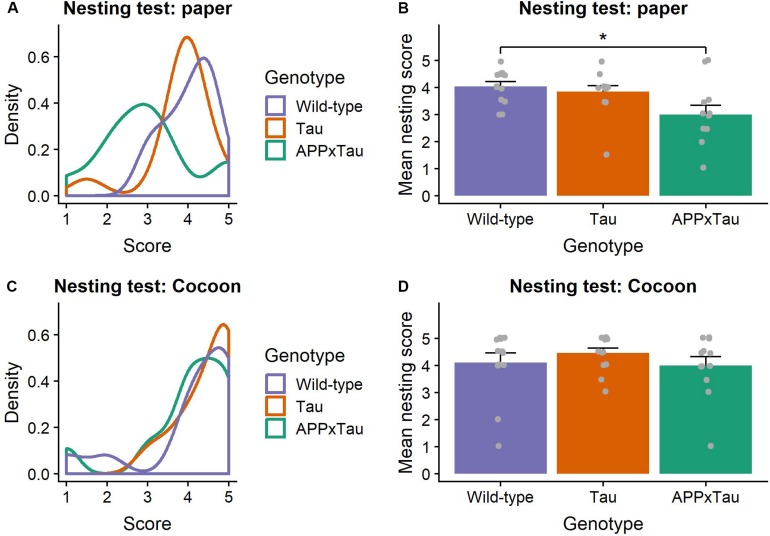
Nesting scores. **(A)** Density plot of nesting scores in the first nesting task. The distribution modes largely overlap for Tau and wild-type animals, whereas the mode for APP × Tau is markedly lower. **(B)** Mean nesting score per genotype. Dunn test showed a significant difference between wild-type and APP × Tau (*p* = 0.0253). Individual datapoints are presented on the plot. **(C)** Density plot of nesting in the second nesting task. Unlike in the first nesting test, distributions for all genotypes show considerable overlap. **(D)** Mean nesting score for the nesting task with Cocoon. A Kruskal–Wallis ANOVA was non-significant, indicating no difference in nesting score between groups (*p* = 0.5069). Individual datapoints are presented on the plot. Data are presented as mean + the standard error of mean. ^∗^*p* < 0.05.

Visual inspection of the nesting data suggested an unequal distribution of the scores over the different genotypes, with higher scores for wild-type and Tau mice, and on average lower scores for APPxTau (Figure 2A). A non-parametric Kruskal–Wallis ANOVA revealed significant differences between the genotypes (χ^2^ = 7.7956, *p* = 0.0203). *Post hoc* Dunn testing showed a significant difference between wild-type and APP × Tau (*p* = 0.0253), but not between wild-type and Tau, nor between Tau and APP × Tau (Figure 2B). In contrast to the first nesting task, the scores per group were more equally distributed in the second nesting task (Figure 2C). A Kruskal–Wallis test indicated no difference in nesting score between groups (χ^2^ = 1.3588, *p* = 0.5069; Figure 2D).

### Exploration and Anxiety Assessment

#### Elevated Plus Maze

**FIGURE 3:**
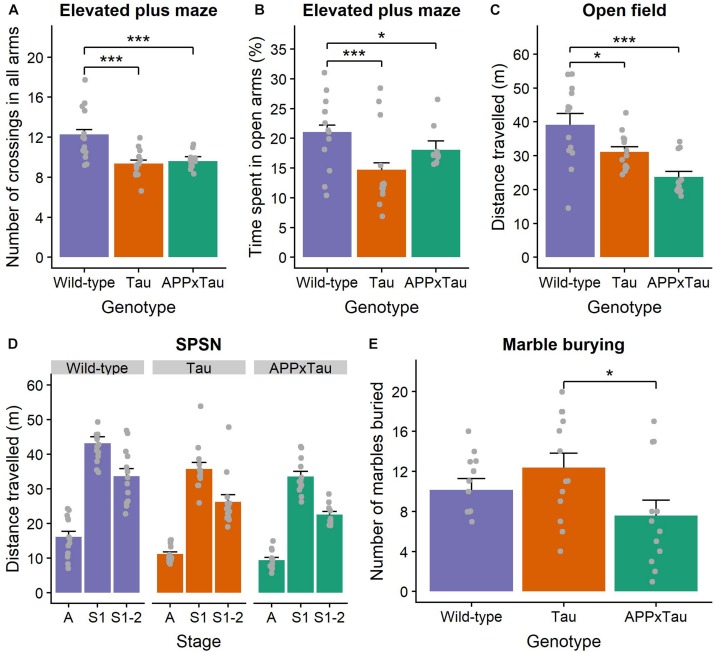
Exploration and anxiety assessment. **(A)** Number of beam crossings in the elevated plus maze was higher for wild-type than transgenic mice. **(B)** Percentage of time spent in the open arms per genotype, with wild-type mice spending significantly more time in the open arms than APP × Tau and Tau mice (*p* = 0.0203 and *p* < 0.001). **(C)** Total distance traveled in the open field differed significantly between genotypes (*p* < 0.001): on average, wild-type animals explored the arena significantly more than Tau and APP × Tau mice. **(D)** Total distance traveled in the SPSN arena was significantly affected by genotype for each stage [*p* = 0.001 for the acclimation (A), sociability (S1), and social novelty (S2) stage]. **(E)** Marble burying scores per genotype. There is a marginally significant difference between APP × Tau and Tau mice (*p* = 0.049). Data are presented as mean + the standard error of mean. A = acclimation phase, S1 = sociability phase; S1-2 = preference for social novelty phase. ^∗^*p* < 0.05; ^∗∗∗^*p* < 0.001.

We found a significant effect of genotype on total number of beam crossings in the elevated plus maze (χ^2^ = 27.323, *p* < 0.001) (Figure 3A). More specifically, a *post hoc* Dunn test revealed a significant difference between wild-type and APP × Tau (*p* < 0.001), and wild-type and Tau animals (*p* < 0.001): wild-type animals were more active overall. The percentage of time spent in the open arms was also significantly influenced by genotype (χ^2^ = 19.11, *p* < 0.001), with wild-type animals spending significantly more time in the open arms than APP × Tau (*p* = 0.0203) and Tau animals (*p* < 0.001) (Figure 3B).

#### Open Field

For the open field task, several parameters were of interest. First, there was a significant genotype effect in the total distance traveled (*F*_2_,_35_ = 10.8438, *p* < 0.001). Tukey tests revealed a specific difference between wild-type and Tau and between wild-type and APP × Tau (*p* = 0.048 and *p* < 0.001, respectively). Thus, wild-type animals explored the arena significantly more than Tau and APP × Tau mice (Figure 3C). The average distance from the center also differed significantly between the genotypes (*F*_2_,_35_ = 3.3056, *p* = 0.048). Tukey tests indicated a significant difference between wild-type and Tau genotypes, with wild-type animals generally being further away from the center than Tau mice (*p* = 0.038, figure not included). We found no genotype effects in the number of entries, time spent, and percentage distance traveled in the center zone (*F*_2_,_35_ = 2.9436, *p* = 0.066; *F*_2_,_35_ = 1.8842, *p* = 0.167; *F*_2_,_35_ = 2.783, *p* = 0.0756) (Supplementary Figure S3A).

Considering the corner zones, there was a significant effect of genotype on the number of entries (*F*_2_,_35_ = 10.1278, *p* < 0.001). A Tukey test indicated a significant difference between wild-type and APPxTau (*p* < 0.001): wild-type mice entered the corner zones significantly more often. The effect of genotype on time spent and percentage distance traveled in the corner zones was non-significant (*F*_2_,_35_ = 1.3526, *p* = 0.272; *F*_2_,_35_ = 2.047, *p* = 0.144) (Supplementary Figure S3B).

Finally, there was a significant effect of genotype on the number of entries to the periphery (*F*_2_,_35_ = 9.4205, *p* = 0.001). According to the Tukey test, wild-type and Tau genotypes entered the periphery significantly more than APP × Tau mice (*p* = 0.002 and *p* = 0.001, respectively). Time spent in the periphery also differed significantly depending on the genotype (*F*_2_,_35_ = 4.5871, *p* = 0.017): wild-type animals spent more time in the periphery than Tau mice (*p* = 0.013). There was a main effect of genotype on the percentage distance traveled in the periphery (*F*_2_,_35_ = 8.192, *p* = 0.001): on average, Tau mice had a lower percentage distance traveled than wild-type and APP × Tau mice (*p* = 0.001; *p* = 0.025) (Supplementary Figure S3C).

#### SPSN

There was a main effect of genotype on the total distance traveled in the arena during the 5-min acclimation phase of the SPSN (*F*_2_,_35_ = 9.1428, *p* = 0.001). The *post hoc* Tukey test showed that wild-type mice traveled significantly further than APP × Tau and Tau mice (*p* = 0.001 and *p* = 0.010, respectively). During the 10-minute sociability phase, there was again a main effect of genotype on the distance traveled (*F*_2_,_35_ = 7.9311, *p* = 0.001): wild-type animals traveled further than APP × Tau and Tau mice (*p* = 0.002 and *p* = 0.015, respectively). This main effect of genotype was also present in the social novelty phase (*F*_2_,_35_ = 8.7778, *p* = 0.001). Wild-type animals again traveled a significantly longer distance than APP × Tau and Tau animals (*p* = 0.001 and *p* = 0.020, respectively) (Figure 3D).

Overall, a linear mixed-effects model revealed a main effect of phase (*F*_1_,_102_ = 4.4766, *p* = 0.0368) and an interaction effect between phase and chamber (*F*_1_,_102_ = 7.6612, *p* = 0.0067) on the time spent sniffing. There was no main effect of genotype or chamber on the time spent sniffing in the sociability phase. However, their interaction was significant (*F*_2_,_34_ = 4.4157, *p* = 0.0197), indicating that Tau animals spent more time sniffing the empty cage than the stranger 1 cage, whereas wild-type and APP × Tau mice spent more time sniffing the stranger 1 cage. In the social novelty phase, the chamber significantly influenced time spent sniffing (*F*_1_,_34_ = 4.9710, *p* = 0.0325), with all animals showing a stronger preference for the stranger 2 mouse (*p* = 0.0267) (Supplementary Figure S4).

#### Marble Burying

For the marble burying test, inspection of the means revealed small differences in number of marbles buried for APP × Tau mice (*M* = 7.5833, *SD* = 5.3676) when compared to Tau (*M* = 12.3846, *SD* = 5.1565) and wild-type mice (*M* = 10.1538, *SD* = 4.0793) (Figure 3E). However, the overall genotype difference was marginally not significant (*F*_2_,_35_ = 3.013, *p* = 0.062). A Tukey test revealed a difference between APP × Tau and Tau mice (*p* = 0.049), with Tau mice burying significantly more marbles, but no significant differences in other pairwise comparisons. Remarkably, in contrast to the scores of Tau and wild-type animals, the marble burying scores in APP × Tau mice seemed to be bimodally distributed. Therefore, the effect of rearing environment, more specifically the cage in which animals were housed since weaning was checked as a covariate, but it did not influence the scores.

### Depressive-Like Behavior

For the data analysis of the tail suspension test, the complete recorded trial was divided into two segments of 180 s. There was no effect of genotype, nor of test segment on the total time of immobility (*F*_2_,_35_ = 0.8975, *p* = 0.417 and *F*_1_,_35_ = 2.9409, *p* = 0.095). The main effect of genotype on the number of immobile episodes was non-significant (*F*_2_,_35_ = 2.4494, *p* = 0.101). There were no main effects of genotype or test segment on distance from the center (*F*_2_,_35_ = 0.1992, *p* = 0.820; *F*_1_,_35_ = 3.6203, *p* = 0.065), nor was there a significant effect of genotype on latency to the first immobile episode (*F*_2_,_35_ = 0.2047, *p* = 0.816) (Supplementary Figure S5).

### Spatial Learning and Memory

**FIGURE 4:**
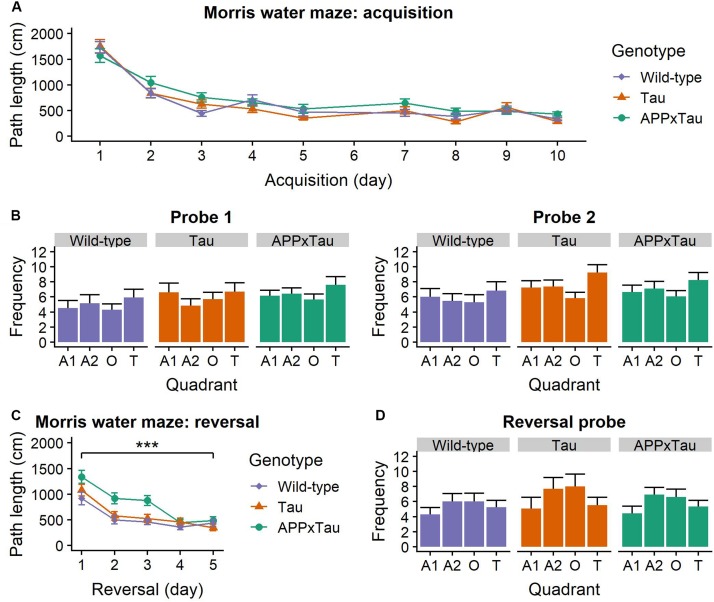
Performance in the Morris Water Maze. **(A)** Average acquisition path length. There was a main effect of acquisition day (*p* < 0.001), but no effect of genotype. **(B)** Number of entries to each quadrant during the probe trials. For both trials, there was a main effect of quadrant (*p* = 0.0064 and *p* < 0.001, respectively). **(C)** Average reversal path length was significantly affected by genotype (*p* = 0.0004) and reversal day (*p* < 0.001): path length became shorter over time. APP × Tau animals had a significantly longer path length than Tau (*p* = 0.0006) and wild-type animals (*p* < 0.001). **(D)** Number of entries in each quadrant during the reversal probe was also significantly influenced by quadrant (*p* < 0.001). Data are presented as mean + the standard error of mean. A1 = adjacent 1 quadrant; A2 = adjacent 2 quadrant, O = opposite quadrant; T = target quadrant. ^∗∗∗^*p* < 0.001.

A linear mixed-effects model indicated a significant main effect of acquisition day on the average path length (*F*_1_,_1285__.__73_ = 312.211, *p* < 0.001) in the Morris water maze. *Post hoc t*-testing showed a significant difference between the first and second acquisition week: path length was shorter in the second week (*t* = 12.129, *p* < 0.001) (Figure 4A). This significant decrease in path length over time is indicative of successful learning of the platform position for all mice.

For the probe trials, number of entries in the quadrants was affected by the quadrant (*F*_3_,_397_ = 8.8036, *p* < 0.001) and probe day × quadrant interaction (*F*_3_,_397_ = 8.7868, *p* < 0.001) (Figure 4B). Consequently, a linear mixed-effects model was applied separately for the first and the second probe day. For both probe days, there was a main effect of quadrant on number of entries (*F*_3_,_105_ = 4.3299, *p* = 0.0064 and *F*_3_,_105_ = 10.8484, *p* < 0.001). *Post hoc t*-tests revealed a significant difference between the target and the opposite quadrant (*p* = 0.0222) and the target and the adjacent 1 quadrant (*p* < 0.001), but not between the target and the adjacent 2 quadrant (*p* = 0.1033).

During the third week, the platform was placed in the opposite quadrant as before to assess flexibility of search strategies. Path length was significantly dependent on genotype (*F*_2_,_151__.__95_ = 8.273, *p* = 0.0004) and reversal day (*F*_1_,_719__.__75_ = 117.425, *p* < 0.001): path length gradually became shorter. There was a significant genotype × reversal day interaction (*F*_2_,_719__.__74_ = 4.136, *p* = 0.0164). *Post hoc t*-tests revealed a significantly shorter path length for wild-type than APP × Tau mice (*p* < 0.001) and for Tau compared to APP × Tau mice (*p* < 0.001) (Figure 4C).

A linear mixed-effects model revealed a significant effect of quadrant on the number of entries (*F*_3_,_105_ = 10.7006, *p* < 0.001) during the reversal probe. *Post hoc t*-tests showed a significant difference between the reverse-target and the adjacent 1 quadrant (*p* < 0.001), the reverse-target and the adjacent 2 quadrant (*p* = 0.0009), and the reverse-target and the opposite quadrant (*p* = 0.0129) (Figure 4D). There was no effect of genotype on the number of entries in the quadrants.

Two important considerations need to be taken into account when interpreting these Morris water maze results. First of all, on top of the main effects described, we also found many interaction effects, which make it more difficult to interpret these main effects. Second, four animals, of which three wild-types and one Tau, displayed severe floating behavior, defined as spending at least 10% of the trial inactively floating on the water. These animals were treated as outliers and removed from additional analyses for the probe trials, but the results remained largely similar.

### Aversive Learning and Pain Perception

#### Fear Conditioning

**FIGURE 5:**
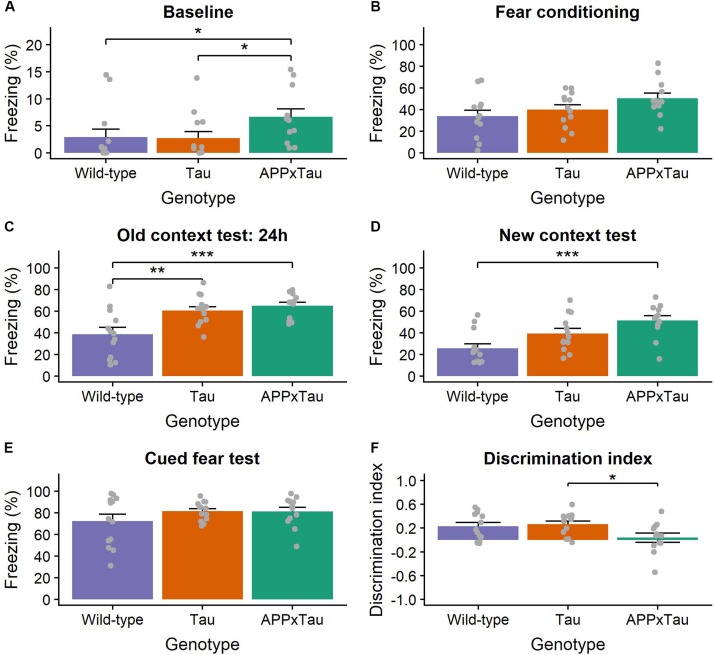
Percentage of freezing during the context- and cue-dependent fear conditioning. **(A)** During the baseline of the fear conditioning phase, transgenic mice showed more freezing than wild-types (*p* = 0.0134). **(B)** There was, however, no difference during the conditioning phase. **(C)** In line with our expectations, all animals showed increased freezing during the old context test 24 h later. Transgenic mice, however, again showed increased freezing (*p* < 0.001). **(D)** Transgenic mice, more specifically APP × Tau, showed significantly more freezing during the new context test (*p* = 0.0106), **(E)** whereas there was no effect of genotype during the cued fear test. **(F)** The discrimination index, used as a quantification of fear memory generalization, was significantly lower in transgenic, especially APP × Tau mice, than in wild-types (*p* = 0.0406). Data are presented as mean + the standard error of mean. Note that the *y*-axis for subpanel A is scaled differently. ^∗^*p* < 0.05; ^∗∗^*p* < 0.01; ^∗∗∗^*p* < 0.001.

A linear mixed-effects model revealed a significant effect of genotype, stage, and the interaction between genotype and stage on percentage freezing during fear conditioning (*F*_2_,_35__.__103_ = 8.845, *p* < 0.001, *F*_3_,_104__.__297_ = 256.612, *p* < 0.001, and *F*_6_,_104__.__295_ = 4.082, *p* = 0.0010, respectively). *Post hoc t*-tests indicated a significantly higher level of freezing during context test and cued fear test than during fear conditioning (both *p* < 0.001). There was a significant effect of genotype on the percentage freezing during baseline (χ^2^ = 8.6248, *p* = 0.0134), the context test (*F*_2_,_35_ = 9.144, *p* < 0.001), and the cued fear test (*F*_2_,_35_ = 5.188, *p* = 0.0106). More specifically, there was a significant difference between wild-type and APP × Tau as well as between Tau and APP × Tau during baseline (*p* = 0.0277 and *p* = 0.0357), between wild-type and Tau and APP × Tau during the context test (*p* = 0.0055 and *p* < 0.001), and between wild-type and APP × Tau in the new context (*p* < 0.001) (Figures 5A–E). There was also a significant effect of genotype on discrimination index (*F*_2_,_35_ = 3.516, *p* = 0.0406), more specifically, a *post hoc t*-test revealed a difference between Tau and APP × Tau mice (*p* = 0.0464) (Figure 5F).

The linear mixed-effects model also indicated a significant effect of genotype, stage, and the interaction between genotype and stage on percentage freezing during the retention phase (*F*_2_,_35_ = 25.8108, *p* < 0.001, *F*_1_,_35_ = 13.4432, *p* < 0.001, and *F*_2_,_35_ = 5.1106, *p* = 0.0113, respectively). There was a significant effect of genotype on the percentage freezing during the context test (*F*_2_,_35_ = 21.05, *p* < 0.001), the new context (χ^2^ = 14.97, *p* < 0.001), and the cued fear test (*F*_2_,_35_ = 5.753, *p* = 0.0069). Again, wild-type animals showed a lower percentage of freezing than APP × Tau and Tau mice for all test phases (*p* < 0.001 and *p* < 0.001 for the old context, *p* < 0.001 and *p* = 0.0167 for the new context, and *p* = 0.0122 and *p* = 0.0217 for the cued fear test for APP × Tau and Tau mice, respectively). There was, however, no significant difference in discrimination index during the retention phase (*p* = 0.6919) (Supplementary Figure S6).

#### Tail Withdrawal Test

Finally, reaction to thermal nociceptive stimuli was operationalized as the latency to tail withdrawal in hot water. There was an expected significant effect of temperature (*F*_3_,_105_ = 49.439, *p* < 0.001): higher temperatures led to faster tail withdrawal (Supplementary Figure S7). There was no effect of genotype on the latency of tail withdrawal (*F*_2_,_35_ = 0.8322, *p* = 0.4435), nor an interaction effect between genotype and temperature (*F*_6_,_105_ = 1.697, *p* = 0.1288). Additionally, possible genotype differences were tested for each temperature, but were non-significant as well (*F*_2_,_35_ = 3.166, *p* = 0.0545; *F*_2_,_35_ = 0.717, *p* = 0.495; *F*_2_,_35_ = 1.49, *p* = 0.239, and *F*_2_,_35_ = 0.974, *p* = 0.388 for 47, 49, 51, and 53°C, respectively).

## Discussion

The present study aimed to identify early behavioral and cognitive deficits in two transgenic mouse models of tauopathy and AD, respectively, more specifically Tau.P301L and APP.V717I × Tau.P301L mice. To achieve this goal, the performance of transgenic mice in several behavioral and cognitive tasks was compared to that of C57BL/6J wild-type animals at the age of three months. All tests and results are summarized in Table 1.

**TABLE 1:** Overview of the results in transgenic compared to wild-type mice for the different behavioral tasks.

	**Tau**	**APP × Tau**	**Age (weeks)**	**Figures**
**General neuromotor assessment**				
Spontaneous 23 h activity Rotarod	= =	↓^∗∗^ =	11 13	1A 1B
**Natural murine behavior**				
Nesting 1 Nesting 2	= =	↓^∗^ =	12 19	2A 2B
**Exploration and anxiety assessment**				
Elevated plus maze: number of crossings; % time spent in open arms	↓^∗∗∗^ ↓^∗∗∗^	↓^∗∗∗^ ↓^∗^	13 13	3A 3B
Open field: distance traveled; distance from center number of entries to center time spent in center % distance traveled in center number of entries to corner time spent in corner % distance traveled in corner number of entries to periphery time spent in periphery % distance traveled in periphery	↓^∗^ ↓^∗^ = = = = = = = ↓^∗^ ↓^∗∗^	↓^∗∗∗^ = = = = ↓^∗∗∗^ = = ↓^∗∗^ = =	14 14 14 14 14 14 14 14 14 14 14	3C X S3A S3A S3A S3B S3B S3B S3C S3C S3C
SPSN: distance traveled in acclimation phase distance traveled in sociability phase distance traveled in social novelty phase time sniffing	↓^∗^ ↓^∗^ ↓^∗^ =	↓^∗∗^ ↓^∗∗^ ↓^∗∗^ =	14 14 14 14	3D 3D 3D S4
Marble burying	=	=	15	3E
**Depressive-like behavior**				
Tail suspension	=	=	15	S5
**Spatial learning and memory**				
MWM: acquisition; acquisition probe trial; reversal; reversal probe trial	= ↓^∗∗^ = =	= ↓^∗^ ↑^∗∗∗^ =	16 17–18 18 19	4A 4B 4C 4D
**Aversive conditioning and pain perception**				
CFR: baseline; fear conditioning; old context test; new context test; cued fear test; discrimination index; old context retention; new context retention; cue retention; discrimination index retention	= = ↑^∗∗^ = = = ↑^∗∗∗^ ↑^∗^ ↑^∗^ =	↑^∗^ = ↑^∗∗∗^ ↑^∗∗∗^ = = ↑^∗∗∗^ ↑^∗∗∗^ ↑^∗^ =	20 20 20 20 20 20 23 23 23 23	5A 5B 5C 5D 5E 5F S6A S6B S6C S6D
Tail withdrawal	=	=	21	S7

*^∗^*p* < 0.05; ^∗∗^*p* < 0.01; ^∗∗∗^*p* < 0.001.*

Preclinical AD and MCI are mainly characterized by cognitive symptoms (Bateman et al., 2012) and motor skills are often affected in later stages of the disease. Nonetheless, exploration and motor activity were assessed in several behavioral tasks. In the spontaneous activity task, all animals showed the expected first exploratory peak and three nocturnal peaks. However, the first two peaks were less pronounced in APP × Tau and Tau animals than for wild-type animals, indicating less exploratory behavior. This was corroborated by the fact that transgenic mice were less actively exploring the elevated plus maze in comparison to wild-type mice, even at an early age. Furthermore, they traveled less distance in the open field and during all SPSN phases. The robust reduction of exploratory behavior in transgenic mice was probably not due to impaired motor coordination, considering their intact rotarod performance, consistent with a previous report on the original Tau-P301L line in FVB/N background (Terwel et al., 2005).

Two nesting tasks were used to assess natural murine behavior, which can be translated to ADLs in humans (Deacon, 2012). ADLs like bathing, dressing oneself, and eating are typically affected in later stages of AD (Alzheimer’s Association, 2016), although subtle differences in IADLs might already present up to 10 years before diagnosis (Fuentes, 2012). In the first nesting task, using regular paper towels as nesting material, APP × Tau mice scored significantly worse than Tau and wild-type mice. However, this finding was not reproduced in the second nesting task, using Cocoon nestlets. Although mice have shown strong preferences for and increased nesting behavior with certain nesting materials over others (Van de Weerd et al., 1997), an immediate comparison between paper and Cocoon has not been made. Therefore, it is unclear if preference for nesting material contributes to the difference in results between the first and second nesting test. If Cocoon is preferred over paper, this could explain the increased nesting behavior and quality of nests in all groups. However, the results could also be influenced by a difference in ease of use between the nesting materials. We hypothesize that making nests with “suboptimal” material like paper is more difficult, and therefore more relying on intact motor coordination and activity level, of which the latter was affected in the transgenic mice in this study. The density and structure of the Cocoon nestlets might have allowed for easier building of high-quality nests, thereby relying less on time, motivation, and activity of the mice.

In general, preclinical AD is characterized by an increased prevalence of late-life psychiatric symptoms, especially anxiety and mood disorders (Steenland et al., 2012). Consequently, this study specifically assessed anxiety- and depression-like behavior in mice. Transgenic mice spent significantly less time in the open arms of the elevated plus maze than age-matched control animals, which is indicative of increased anxiety in these transgenic mice. Nevertheless, this was not confirmed in the open field task, where wild-type animals generally stayed further away from the center than transgenic mice, suggesting the opposite. Digging activity and anxiety-like behavior, assessed with marble burying, did not seem altered in transgenic mice either, as there was no significant difference in marble burying scores between genotypes. Together, these findings do not provide conclusive evidence of an overall increase in anxiety-related behavior in transgenic mice as compared to controls. However, these different tasks tap into different domains of anxiety-like behavior, and therefore it is not surprising that only one domain is affected during this early pathology stage. Depression-like behavior, reflected by the tail suspension test, did not differ between wild-type and transgenic mice either for any of the parameters tested.

Alzheimer’s disease is often characterized by social withdrawal in both humans and mice (Filali et al., 2011) and this social withdrawal is already present before AD diagnosis (Jost and Grossberg, 1996). In the SPSN sociability stage, Tau mice spent significantly more time sniffing the empty cage than the cage with the stranger 1 mouse, while the reverse was true for APP × Tau and wild-type mice. During the social novelty stage, all mice spent more time sniffing the novel mouse (stranger 2) than stranger 1. There was no difference in the time spent inside the chambers, indicating that all mice spent a similar amount of time with the stranger mice. Thus, wild-type and transgenic mice did not differ in their preference for social novelty, but Tau mice showed a decreased sociability compared to APP × Tau and wild-type mice.

Deficits in episodic memory, typically caused by pathology in the hippocampus and rest of the temporal lobe, arise very early and are a key characteristic of preclinical AD (Webster et al., 2014). In mice, deficits in hippocampus-dependent spatial learning and memory typically emerge after 3–6 months, depending on the mouse model (Webster et al., 2014). Consequently, reference and working memory were assessed using the MWM. Overall, there was no difference between genotypes in average path length during the acquisition phase, reflecting intact spatial learning. Surprisingly, transgenic mice entered the target quadrant more often than wild-type mice during the probe trials, although it is important to note that they entered *all* quadrants more often. During the reversal trials, which reflect a form of cognitive flexibility, wild-type animals had a shorter path length than transgenic mice. These findings indicate that transgenic were less flexible in their search strategy than wild-type mice, corresponding with the early deficits in executive functioning observed in humans (Bäckman et al., 2005). Interestingly, APP × Tau animals had a significantly longer path length during the reversal than Tau (*p* = 0.0006) and wild-type animals (*p* < 0.000) which could indicate a more severe deficit in this measure due to the interaction of APP- and Tau-mediated pathology.

Finally, aversive learning was assessed using the context- and cue-dependent fear conditioning protocol (Paradee et al., 1999). Overall, transgenic mice displayed a higher level of freezing than control mice during the conditioning phase. This pattern of increased freezing was also present in the retention phase, 21 days later. The missing genotype differences in the tail withdrawal test, excluded differences in pain perception as being causative for the increased freezing of transgenic mice. Consequently and in line with a study by Wang et al. (2004), contextual and cued fear learning was not impaired in transgenic mice. On the other hand and in line with our results from the elevated plus maze, these findings could indicate increased anxiety in transgenic mice.

Previous research found differences in cognitive performance between transgenic Tau mouse models and transgenic APP models (Lo et al., 2013). In the current study, we compared Tau mice with APP × Tau mice, to investigate the possible harmful contribution of human amyloid to tau pathology in early disease stages. Our results revealed three domains in which APP × Tau mice performed worse than Tau mice: (i) decreased nesting behavior in the nesting task with paper, (ii) decreased discrimination index during aversive learning, and (iii) they had a significantly longer path length than Tau and wild-type animals in the reversal phase of the water maze task which is pointing to a more severe deficit in cognitive flexibility. These results are not surprising, as the presence of Aβ oligomers in APP × Tau is thought to accelerate tau pathology (Selkoe and Hardy, 2016) and cognitive impairment (Spires-Jones and Hyman, 2014; Webster et al., 2014). In sum, our results are in apparent agreement with the amyloid cascade hypothesis.

Mouse models have been developed for a wide variety of diseases, including AD. The variety of generated AD mouse models has been invaluable to delineate distinct pathological mechanisms and the resulting physiological and behavioral consequences. Nonetheless, there are some limitations to this study and to the use of animal models in general. First, we used only male mice and thus could not investigate potential gender effects, which have been observed in other AD mouse models and in patients (Fisher et al., 2018). This was both for practical reasons and because this allowed a better comparison to previous reports on these models which also studied only male mice (Boekhoorn et al., 2006; Chong et al., 2011). Nevertheless, in another study using female mice of these models at the same age, we have observed exactly the same phenotypes in the contextual fear conditioning paradigm, suggesting that—at least at this early disease stage—there are no overt sex differences in cognitive (dys)function (unpublished findings). Second, mice do not display the full range of human behavior and thus cannot model all aspects of human cognition, social behavior and disease. Moreover, amyloid and tau pathology may act differently in mice compared to humans, and different tau substrains may be involved in different tauopathies, a topic that has only recently become a focus of research. Transgenic models are also prone to artifacts due to unphysiological overexpression systems (Jankowsky and Zheng, 2017). Nonetheless, despite these limitations, behavioral testing in rodents remains relevant for human AD and future models that are based on improved understanding of the multiple causes of SAD, hold great promise for AD research (Onos et al., 2016).

Mouse models should always be selected with careful consideration of the specific research questions and goals of the study (Jankowsky and Zheng, 2017). It would be interesting to repeat the current study using other transgenic as well as knock-in mouse models. APP knock-in mouse models, for example, show amyloid pathology without overexpression of APP (Saito et al., 2014). The more physiological APP expression patterns may offer a closer recapitulation of human AD, and reduce experimental artifacts that would lead to wrong conclusions (Latif-Hernandez et al., 2017; Sasaguri et al., 2017). In general, it is difficult to directly compare different transgenic mouse strains (Götz et al., 2004), yet, future research should try to replicate the current findings in other mouse models in order to develop a robust taxonomy of preclinical behavioral and social changes in mice.

## Conclusion

This study uncovered early differences in natural murine behavior, exploratory behavior, anxiety-like behavior, and fear learning, as well as inflexibility in hippocampus-dependent learning in two mouse models that reproduce certain pathological features of human AD. These findings can be indicative of small but significant changes that are present in older adults with preclinical AD as well. Further research is necessary to identify clear behavioral markers of preclinical AD that are applicable in clinical settings.

## Data Availability Statement

The datasets generated for this study are available on request to the corresponding author.

## Ethics Statement

The animal study was reviewed and approved by the Animal Ethics Committee of KU Leuven.

## Author Contributions

AS, DB, and SS conceived and designed the study and provided feedback to the manuscript. CS and SS conducted the experiments. CS analyzed the data and wrote the manuscript. All authors gave approval of the final version and are accountable for this work. The corresponding author attested that all listed authors met authorship criteria and that no others meeting the criteria have been omitted.

## Conflict of Interest

The authors declare that the research was conducted in the absence of any commercial or financial relationships that could be construed as a potential conflict of interest.
