# Supplementary material for: Early Cognitive and Behavioral Deficits in Mouse Models for Tauopathy and Alzheimer’s Disease
Source: Front Aging Neurosci. 2019 Dec 6;11:335. doi: 10.3389/fnagi.2019.00335 (PMC6908963; doi:10.3389/fnagi.2019.00335)
Supplement: Supplementary file 1 [file Data_Sheet_1.PDF]

Supplementary Material

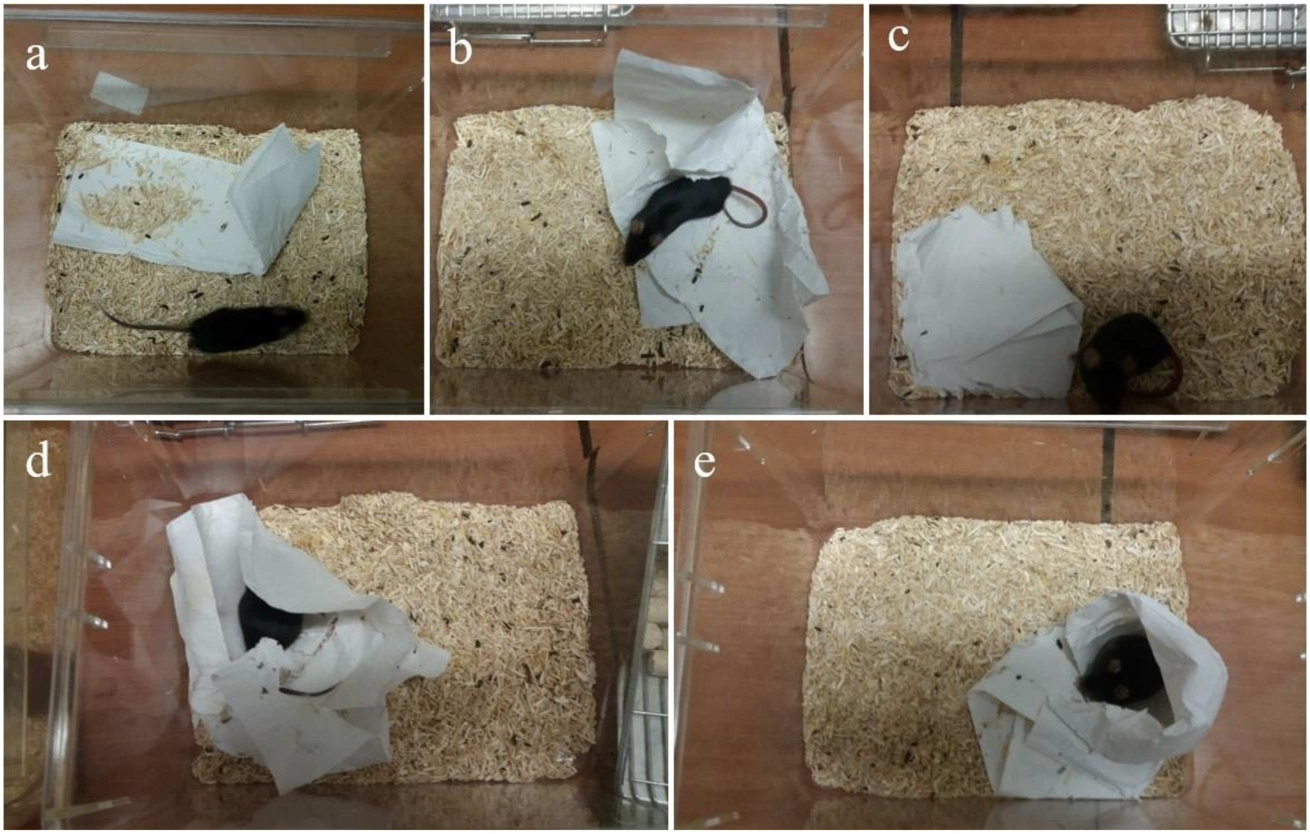

Supplementary Figure 1. Nesting scores on the 5-point rating scale according to Deacon (2012) when provided with a single paper towel as nesting material. **(A)** score 1: the nesting material is largely untouched. **(B)** score 2: the nesting material is only partially torn up. **(C)** score 3: the nesting material is shredded up for more than 50%, but there is no identifiable nest site. **(D)** score 4: there is an identifiable nest site, but the nest is relatively flat. **(E)** score 5: the nest is clearly identifiable and the walls of the nest are higher than the animal's body height.

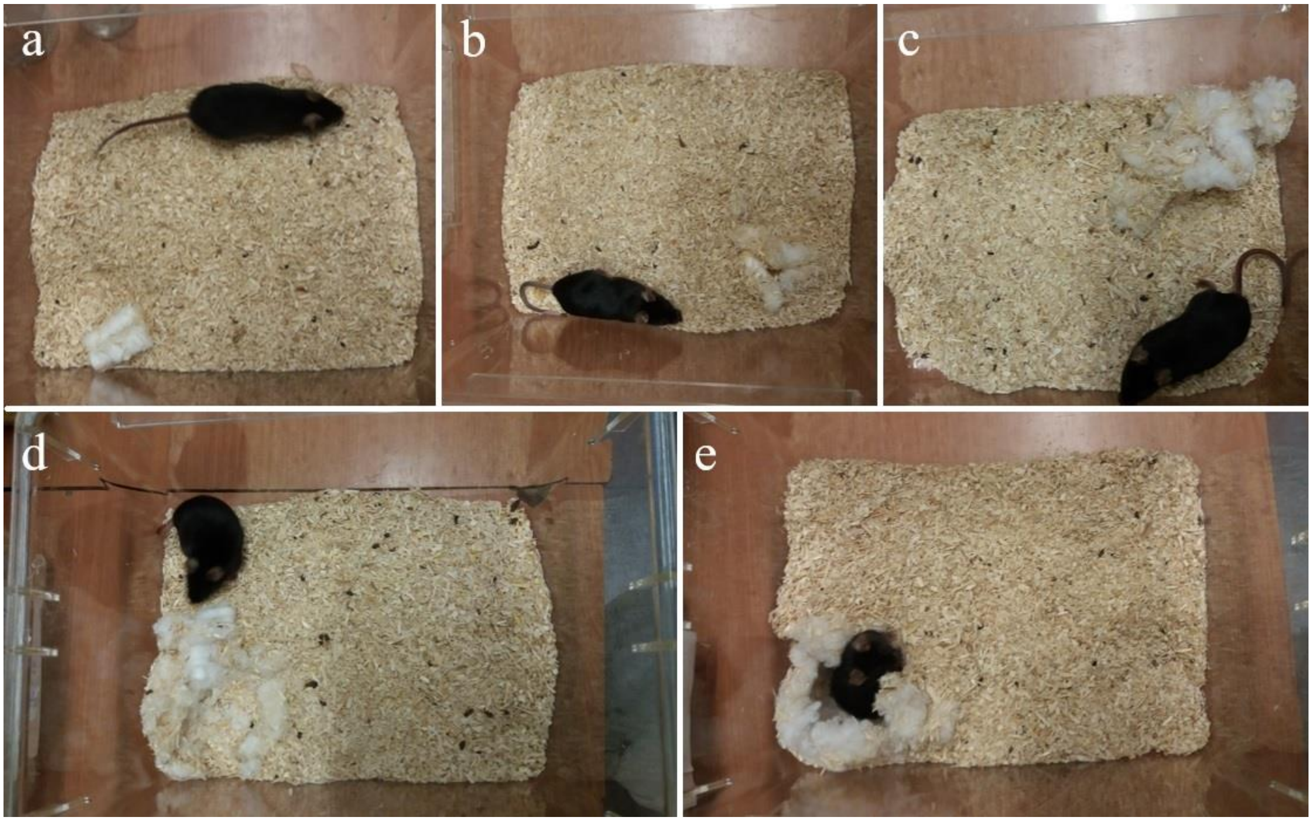

Supplementary Figure 2. Nesting scores on the same 5-point rating scale as in Suppl. Fig.1 but with a Cocoon nestlet provided as nesting material. **(A)** score 1: the nesting material is largely untouched. **(B)** score 2: the nesting material is only partially torn up. **(C)** score 3: the nesting material is shredded up for more than 50%, but there is no identifiable nest site. **(D)** score 4: there is an identifiable nest site, but the nest is relatively flat. **(E)** score 5: the nest is clearly identifiable and the walls of the nest are higher than the animal’s body height.

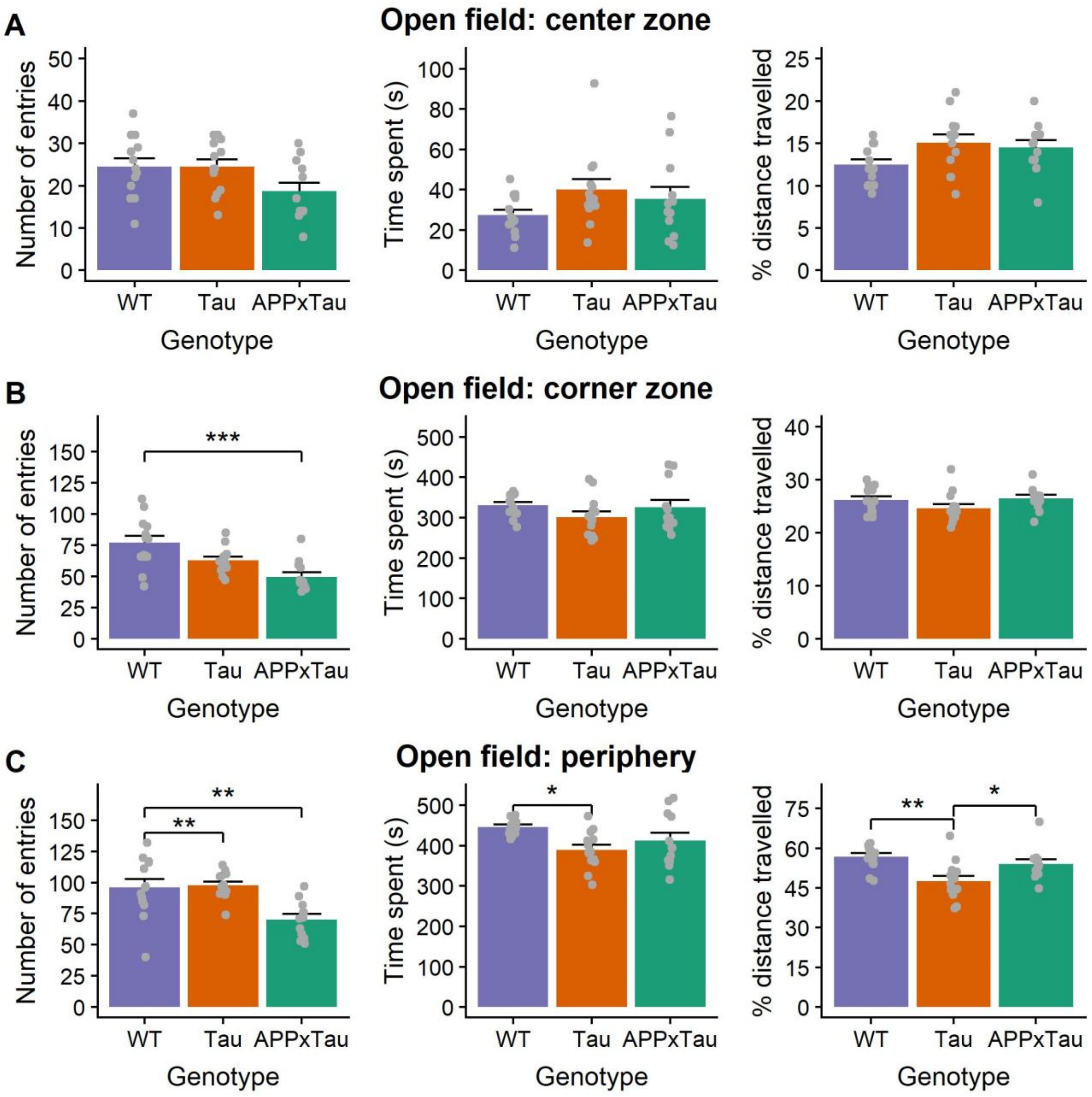

Supplementary Figure 3. Open field test for general exploration and anxiety. (A) Number of entries to, time spent and percentage distance travelled in the center zone. There was no effect of genotype on the number of entries to, time spent and percentage distance travelled in the center zone. **(B)** Number of entries to, time spent and percentage distance travelled in the corner zones. Wild-type animals entered the corner zones significantly more often than APPxTau mice ($p < 0.001$), but there was no effect of genotype on time spent and percentage distance travelled in the corner zones. **(C)** Number of entries to, time spent and percentage distance travelled in the periphery. Wild-type and Tau mice entered the periphery significantly more than APPxTau mice ($p = 0.001$), wild-type animals spent more time in the periphery than Tau mice ($p = 0.017$) and wild-type and APPxTau had a higher percentage of distance travelled than Tau mice ($p = 0.001$; $p = 0.025$). Individual datapoints are presented on the plot. Data are presented as mean + the standard error of mean. Note that not all y-axes are scaled evenly to improve clarity. WT = wild-type. * $p < 0.05$; ** $p < 0.01$; *** $p < 0.001$.

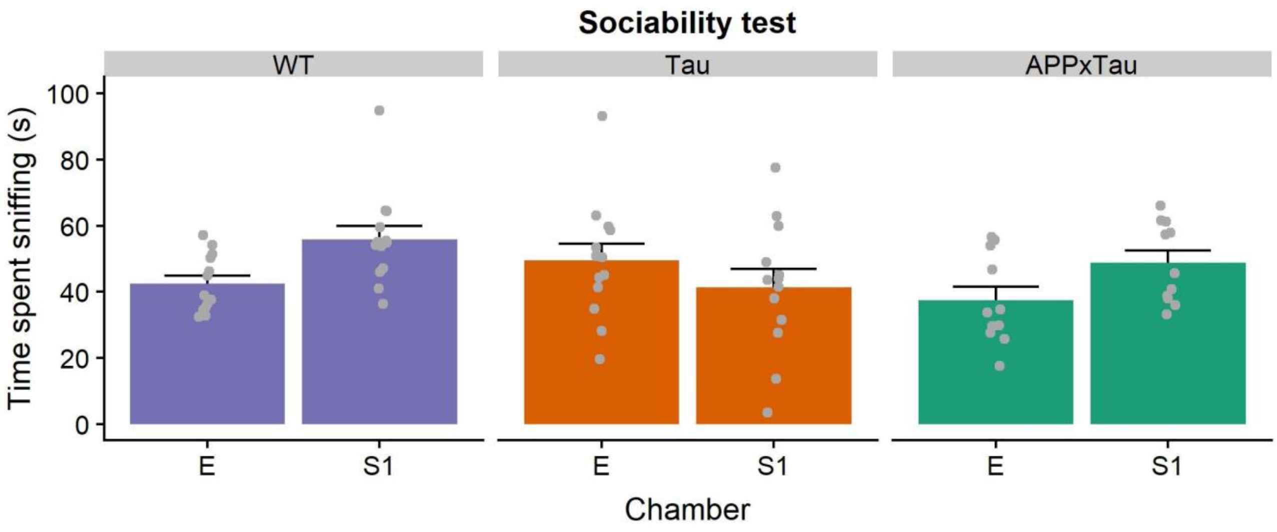

Supplementary Figure 4. Sociability and preference for social novelty (SPSN) test. Overall time spent sniffing during SPSN. There was a significant effect of stage (p = 0.0368) and an interaction effect of stage and chamber (p = 0.0067) on overall time sniffing. There was also a significant genotype x chamber-interaction effect during the sociability phase ($F_{2, 34}$ = 4.4157, p = 0.0197), indicating that, unlike APPxTau and wild-type, Tau animals spent more time sniffing the empty cage than the stranger 1 cage. In the social novelty phase, the chamber significantly influenced time spent sniffing ($F_{1, 34}$ = 4.9710, p = 0.0325), with all animals showing a stronger preference for the stranger 2 mouse (p = 0.0267). Individual datapoints are presented on the plot. Data are presented as mean + the standard error of mean. WT = Wild-type; E = empty cage; S1 = stranger 1 mouse; S2 = stranger 2 mouse.

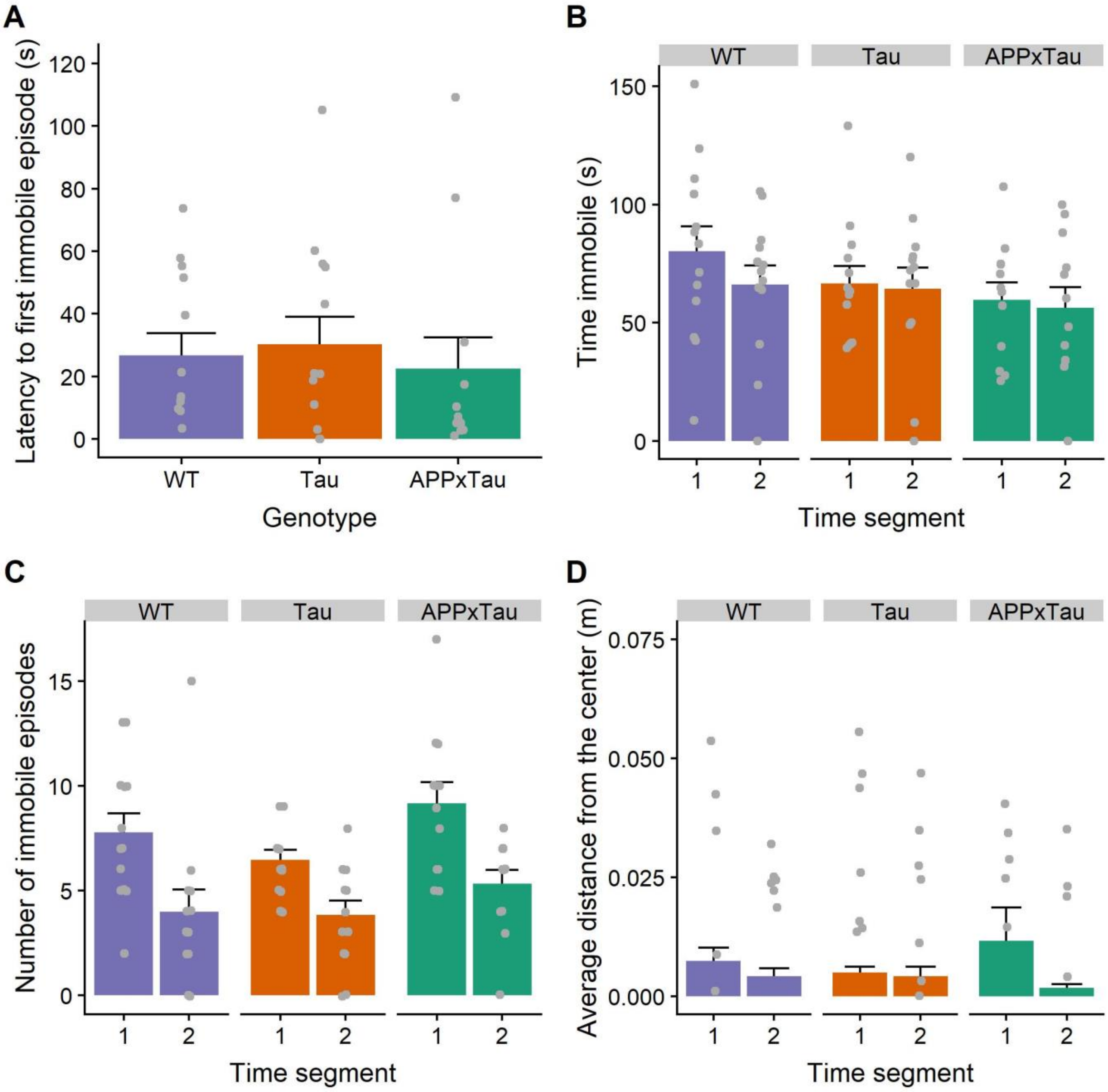

Supplementary Figure 5. Tail suspension test. (A) Latency to first immobile episode during the tail suspension test was independent of genotype. **(B)** The effect of genotype and test segment on the total time immobile was also non-significant. **(C)** Number of immobile episodes were affected by test segment ($p < 0.001$), but not by genotype. **(D)** There were no significant differences in average distance from the center. Individual datapoints are presented on the plot. Data are presented as mean + the standard error of mean. WT = Wild-type; time segment 1 = 0 – 180s; time segment 2 = 180 – 360s.

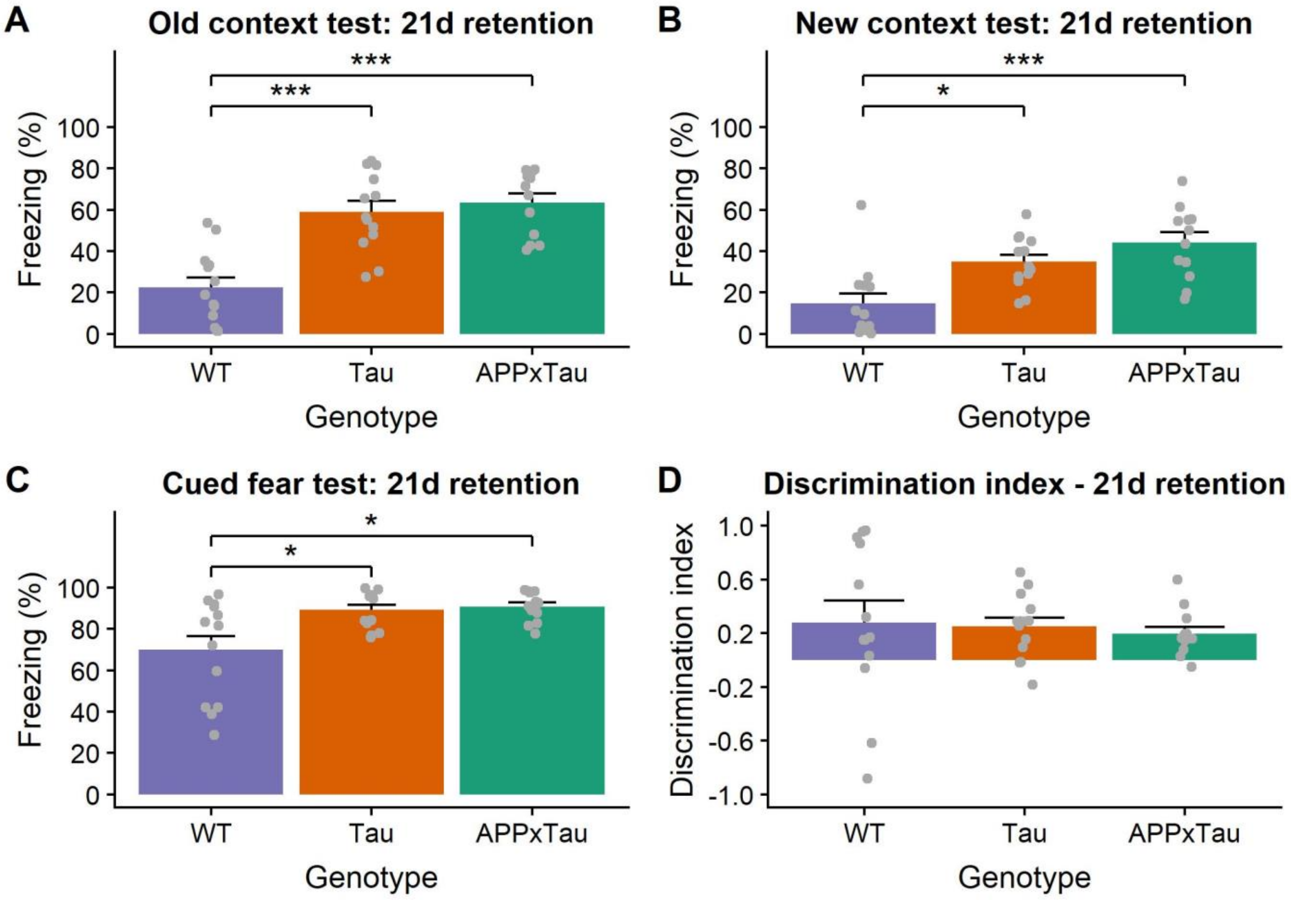

Supplementary Figure 6. Context- and cue-dependent fear conditioning. (A) Transgenic mice displayed higher freezing scores during the old context test after 21 days retention ($p < 0.001$), **(B)** as well as during the new context test ($p = 0.0069$) **(C)** and cued fear test after 21 days ($p < 0.001$). **(D)** There was no effect of genotype on the discrimination index after 21 days retention. Data are presented as mean + the standard error of mean. WT = Wild-type. * $p < 0.05$; *** $p < 0.001$.

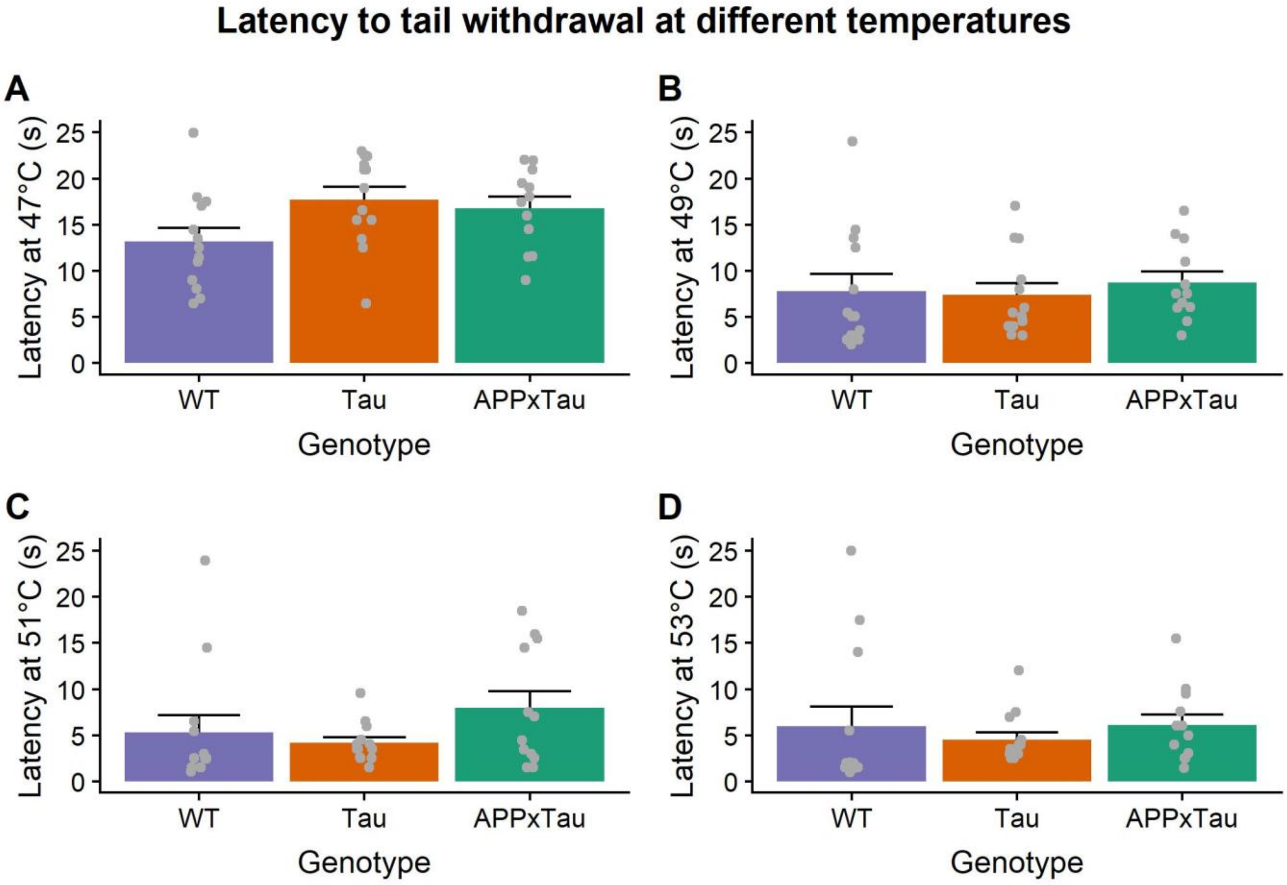

Supplementary Figure 7. Tail withdrawal test. Mean latency to tail withdrawal in **(A)** 47°C, **(B)** 49°C, **(C)** 51°C and **(D)** 53°C water. The barplot shows an expected significant effect of temperature ($p < 0.001$): higher temperatures led to faster tail withdrawal. Data are presented as mean + the standard error of mean. WT = Wild-type.
